# Development of algorithms for estimating the Child Health Utility 9D from Caregiver Priorities and Child Health Index of Life with Disabilities

**DOI:** 10.1007/s11136-024-03661-9

**Published:** 2024-05-03

**Authors:** Utsana Tonmukayakul, Kate Willoughby, Cathrine Mihalopoulos, Dinah Reddihough, Brendan Mulhern, Rob Carter, Suzanne Robinson, Gang Chen

**Affiliations:** 1 Deakin Health Economics, Institute for Health Transformation, Faculty of Health, Deakin University, Geelong, VIC Australia; 2 Orthopaedic Department, The Royal Children’s Hospital, Parkville, Melbourne, VIC Australia; 3 Murdoch Children’s Research Institute, Parkville, Melbourne, VIC Australia; 4 Division of Health Economics, Public Health and Preventive Medicine, Monash University, Melbourne, VIC Australia; 5 Centre of Health Economics Research and Evaluation, University of Technology Sydney, Haymarket, Sydney, NSW Australia; 6 Centre for Health Economics, Monash Business School, Monash University, Melbourne, VIC Australia

**Keywords:** Mapping, Quality-adjusted life years, CPCHILD, CHU9D, Cerebral palsy, Quality of life, Non-ambulatory children

## Abstract

**Purpose:**

The primary aim was to determine Child Health Utility 9D (CHU9D) utilities from the Caregiver Priorities and Child Health Index of Life with Disabilities (CPCHILD) for non-ambulatory children with cerebral palsy (CP).

**Methods:**

One hundred and eight surveys completed by Australian parents/caregivers of children with CP were analysed. Spearman’s coefficients were used to investigate the correlations between the two instruments. Ordinary least square, robust MM-estimator, and generalised linear models (GLM) with four combinations of families and links were developed to estimate CHU9D utilities from either the CPCHILD total score or CPCHILD domains scores. Internal validation was performed using 5-fold cross-validation and random sampling validation. The best performing algorithms were identified based on mean absolute error (MAE), concordance correlation coefficient (CCC), and the difference between predicted and observed means of CHU9D.

**Results:**

Moderate correlations (*ρ* 0.4–0.6) were observed between domains of the CHU9D and CPCHILD instruments. The best performing algorithm when considering the CPCHILD total score was a generalised linear regression (GLM) Gamma family and logit link (MAE = 0.156, CCC = 0.508). Additionally, the GLM Gamma family logit link using CPCHILD comfort and emotion, quality of life, and health domain scores also performed well (MAE = 0.152, CCC = 0.552).

**Conclusion:**

This study established algorithms for estimating CHU9D utilities from CPCHILD scores for non-ambulatory children with CP. The determined algorithms can be valuable for estimating quality-adjusted life years for cost-utility analysis when only the CPCHILD instrument is available. However, further studies with larger sample sizes and external validation are recommended to validate these findings.

**Supplementary Information:**

The online version contains supplementary material available at 10.1007/s11136-024-03661-9.

## Plain English summary

Deciding which healthcare interventions to invest in can be challenging, especially when considering the diverse impacts of different health conditions. For young people with life-long conditions like cerebral palsy (CP) who cannot walk independently, their health impacts differ from those with conditions like asthma. To help in decision-making about where to invest, health economists have developed and employed an outcome metric called “quality-adjusted life years” (QALYs). QALYs are usually measured using questionnaires that assess health-related quality of life and are scored using special mathematical formulas that take into account what the person values in different areas of life that the questionnaire measures. However, these questionnaires are not commonly used in CP research. This study searched for statistical equations to convert scores from the Caregiver Priorities and Child Health Index of Life with Disabilities (CPCHILD) questionnaire, which is a commonly used instrument for children with CP who cannot walk by themselves, into Child Health Utility 9D utilities for QALY calculation. Two proposed equations using CPCHILD total score and selected CPCHILD domain scores can help estimate QALYs in the CP population. This may help in resource planning, when only the CPCHILD score is available.

## Introduction

Quality-adjusted life years (QALYs) are the preferred health outcome metric used by national reimbursement agencies, such as the Pharmaceutical Benefits Advisory Committee (PBAC) in Australia and the National Institute for Health Care Excellence (NICE) in the UK [1, 2]. The key advantage of QALY(s) is their ability to measure both quantity and quality of life, which allows for a comprehensive evaluation of health interventions. QALYs enable policy makers to compare health benefits across various conditions and interventions, facilitating resource allocation decisions within public healthcare systems.

The premise of QALYs is that the number of years lived with a specific health condition is “weighted” for the quality of life associated with that condition during that time. The weights or utilities are usually measured using instruments that assess various health-related quality of life dimensions. The instruments, which are presented in a multiple-choice format, are known as multi-attribute utility instrument (MAUI). Individual’s responses to the MAUIs are then converted by using algorithms into a numeric index bounded by 0 (being death) to 1 (full health) reflecting individual’s preferences with respect to various domains measured by the MAUIs.

Measuring QALYs in children with cerebral palsy (CP) is important because this population needs comprehensive and specialised healthcare services including medical interventions, rehabilitation, assistive devices, and ongoing support. CP is a group of life-long movement and/or posture disorders resulting from injury or insult to the developing foetal or infant brain [3]. A systematic review reported that the medical costs for children with CP worldwide were 10–26 times greater than those for children who are typically developing with a positive relationship between expenditure and the severity of gross motor function [4].

Measuring QALY in children with CP presents challenges due to limitations in available MAUIs. The recently developed CP-specific instrument, the Cerebral Palsy 6 Dimensions (CP-6D), has not yet been validated in children with CP [5]. Moreover, generic instruments, such as Health Utilities Index Mark 3 (HUI-3), the Assessment Quality of Life-4D, and the EQ-5D-3L, may have limited sensitivity in children because they were adapted from instruments for adults [6]. The widely used generic MAUI, i.e. EQ-5D-Y, may not fully capture the multidimensional aspects of health-related quality of life that are specific to children with chronic neuromuscular conditions [7]. The absence of value sets for English version of EQ-5D-Y further restricts its application [8]. While Ryan et al. [9] proposed Child Health Utility 9D (CHU9D) as a superior alternative to EQ-5D-Y for measuring utility values in children with CP due to its better alignment with their experiences and avoidance of extreme values, its routine used in CP studies remains limited.

To facilitate the QALY measurement in children with CP where the valid and reliable non-MAUI instruments such as the Cerebral Palsy Quality of Life questionnaire (CPQoL) and the Caregiver Priorities and Child Health Index of Life with Disabilities (CPCHILD) are more frequently used, there was an attempt to map the CPQoL children version onto CHU9D [10]. However, the algorithms were developed from mostly young participants with mild-to-moderate severities of gross motor function. There is still a lack of QALY measurement for the severe or non-ambulatory group. The aim of this current study was to develop mapping regressions converting scores of the CPCHILD, a specific instrument designed for non-ambulatory children with CP, onto the CHU9D utilities. These regressions will allow the use of the current form of the CPCHILD for QALY estimation when only the CPCHILD is available, aiding economic evaluations and healthcare decision-making in this population.

## Methods

This study followed the ISPOR good practices for mapping studies [11] and the Mapping onto Preference-based Measure Report Standards (MAPS) checklist [12].

### Design and participants

A cross-sectional survey was conducted in 2019–2022, involving parents/caregivers of young people aged 5–18 years with a diagnosis of CP and functioning at Gross Motor Function Classification System (GMFCS) level IV or V, or non-ambulatory levels. In most instances, previous mapping studies, not limited to children with CP, have used convenience sampling approaches, with sample sizes ranging from 60 to 12,967 [13]. Considering the population size and challenges in participant recruitment for CP studies, the current study also employed a convenience sampling approach. The previous mapping study that focused on children with CP had a sample size of 76 [10]. For the current study, a target sample size of 150 participants was determined based on a total of 676 potential participants identified from the Victoria Cerebral Palsy Register (VCPR) with an expected response rate of 15% derived from a previous Australian study [14]. The VCPR is a population-based register of all young people born or living with CP in the Victorian state of Australia.

The study invitation was sent to the total of 732 participants including all eligible parents/caregivers identified from the VCPR and to 56 identified through attendance at orthopaedic outpatient clinics at the Royal Children’s Hospital, Melbourne. Follow-up phone calls were made to 426 potential participants who did not opt out after receiving the invitation. Participants whose child had died, had limited English, were unreachable by phone, or were under complex case management were excluded from the study.

The survey responses were collected and data managed using the secure, web-based Research Electronic Data Capture (REDCap) [15]. Ethics approval was obtained from the Royal Children’s Hospital Human Research Ethics Committee (HREC# 37238) and Deakin University Ethics Committee (Ref 2019-080). Consent was implied by completion of the survey.

### Measures

The CPCHILD is a widely recognised and utilised instrument in the field of CP, measuring the caregiver’s perspectives of health status, function, comfort, well-being, and ease of caregiving for non-ambulatory children aged 5–18 years functioning at level IV and V on the GMFCS [16]. The GMFCS classifies motor function in CP, ranging from level I (independent walking) to level V (children are dependent on wheeled mobility and have limited head and trunk control). The CPCHILD has shown to be one of the strongest outcome measures with the strongest psychometric properties and clinical utility for children with CP [17, 18]. The CPCHILD questionnaire gathers information pertaining to the previous two weeks. It includes 37 items across six domains: (1) Personal care/activities of daily living, (2) Positioning, transferring and mobility, (3) Comfort and emotions, (4) Communication and social interaction, (5) Health, and (6) Overall quality of life. This study used the CPCHILD parent version 5.

The CPCHILD has a complex structure where each of the six domains has different scoring sections. In domains 1 and 2, the scoring sections evaluate the degree of difficulty in performing activities and the level of assistance required. The scores range from 0 (no problem at all) to 6 (not possible) for ‘difficulty’ and from 0 (total assistance) to 3 (independent) ‘level of assistance’. Domain 3 focuses on rating the frequency and intensity of pain or discomfort during certain daily activities, ranging from 0 (none of the time) to 5 (all the time) for ‘frequency’ and from 0 (severe) to 3 (none) for ‘intensity’. Items in domain 4 rate only the level of difficulty, while domain 5 includes questions asking about health resources used, such as doctor and hospital visits (0 for admitted > 7 days to 5 for none), overall health of the child (0 is very poor to 5 is excellent), and number of medications (0 for ≥ 5 medications to 5 for no medication). Domain 6 consists of a single item rating the child’s overall quality of life, ranging from 0 (very poor) to 5 (excellent) [16].

The CPCHILD total score is derived by averaging standardised scores of each item, while the domain scores average standardised item scores of relevant items. The standardised item score is computed from a sum of categorical rating responses of an item divided by the maximum possible raw item score. The CPCHILD manual provides more details about the scoring of CPCHILD [19]. Despite the complex structure of CPCHILD, its scoring should not raise any challenge for mapping.

The CHU9D is a generic MAUI designed for children and adolescents aged 7–17 [20]. Xiong et al. [21] reported that the CHU9D can be used for children between the ages of 5 and 18, with parent-report for children aged 5–7 years and self-report for children aged 8–18 years. It consists of nine questions that assess child’s worry, sadness, pain, fatigue, annoyance, schoolwork, sleep, daily routine, and ability to participate in activities. Caregivers provide their views on their child over the preceding 24 hours using a 5-level response rating, ranging from 0 (no problems) to 5 (severe problem) [22]. The CHU9D can be completed by either children themselves or a proxy [21]. In this study, a proxy-report was used because many non-ambulatory children with CP were unable to complete questionnaires by themselves. The CHU9D utilities were estimated using the Australian adolescent-specific algorithm [23], which generates utilities between − 0.1059 and 1 [24].

### Statistical analysis

Spearman’s correlation coefficients were determined to explore relationship and overlapping domains between the CPCHILD and CHU9D. A correlation coefficient > 0.7 was considered strong correlation, 0.5–0.7 moderate, and < 0.5 weak [25].

There are two ways to undertake mapping studies, either directly or indirect. Direct mapping directly transfers the total score, selected domain scores, or item scores of the source instrument to predict the utilities of the target instrument. Indirect mapping predicts the response ratings of each item of the target measure based on item or domain data of the source instrument. This study employed direct mapping for two main reasons: it does not require a large sample size and the item structure of the CPCHILD is complex.

The ISPOR guidelines suggested that demographic information be included as predictors, so two core models were initially considered: *Predictor set 1*—CPCHILD total score, age, gender, GMFCS level, and self-perceived economic status, and *Predictor set 2—*selected CPCHILD domain scores, age, gender, GMFCS level, and self-perceived economic status. However, the self-perceived economic status was excluded to enhance the applicability of the mapping algorithms, since it is unlikely that other datasets contain this information. Non-statistically significant predictors (*p* > 0.05) were also excluded from the models to achieve the robust combination of predicting variables [26]. Due to our estimated sample size, this study did not consider the mapping algorithm based on CPCHILD item scores.

To investigate the distribution of CHU9D utilities, a Shapiro–Wilk test for normality was conducted. This information informed the choice of regression methods. Three regression techniques were considered: (1) ordinary least square (OLS), a widely used technique in mapping research [27]; (2) robust MM-estimator, a model suitable for small-sample efficiency [28]; and (3) generalised linear model (GLM) with specifying priori distributions. Four combinations of Gaussian and Gamma families, along with log and logit links, were explored. OLS and GLM have been widely employed in previous mapping studies [27].

### Assessment of predictive accuracy

Model performance was assessed using mean absolute error (MAE) and the concordance coefficient correlation (CCC). MAE is a commonly used indicator in mapping studies [27, 29] and calculates the average absolute differences between the observed and predicted CHU9D utilities. On the other hand, CCC [30] measures the agreement between the observed and predicted CHU9D utilities. A smaller MAE and the larger CCC values indicate a better predictive ability of the mapping algorithm. Additionally, the differences between the predicted and observed means of CHU9D were also considered to identify the best and second-best mapping algorithms.

### Model validation

The validation process involved two internal validation approaches due to the absence of an external dataset. The first approach (Validation 1) is a 5-fold internal cross-validation where the full sample was randomly divided into five equal-size sub-groups. In each iteration, four of the five sub-groups served as the “estimation sample” to develop the mapping algorithms, while the remaining sub-group was used as the “validation sample” to assess the performance of the algorithms. This process was simulated five times, with each sub-group serving as both estimation and validation samples. The average MAE and CCC across the five iterations were computed. In the second approach (Validation 2), the mapping algorithms generated from the full sample were tested on two computer-generated random samples. These random samples had sample size of 80 and 50 [31].

All statistical analyses were conducted in STATA SE16 [32].

## Results

### Sample and descriptive data

**Fig. 1:**
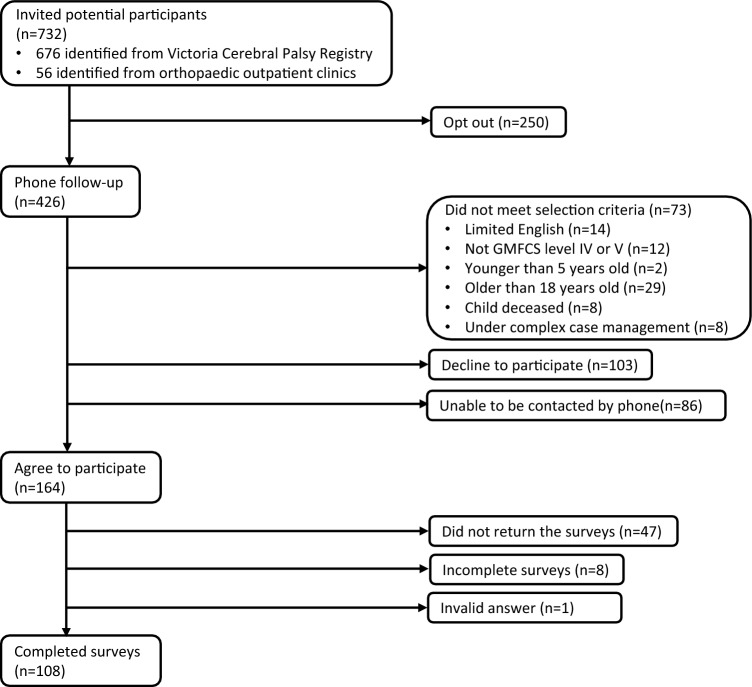
Participant flowchart

The total of 108 participants returned the completed surveys for analysis. Figure 1 provides a flow chart illustrating the recruitment and participation of the participants.

Table 1 presents characteristics of the participants, including their scores on the CPCHILD and CHU9D instruments. The age of the children ranged between 5 and 18 years, with mean age of 12 years. Sixty-one percent were male and 48% functioned at GMFCS level V. The mean CPCHILD total score was 45.38 out of 100. The standardised score of each CPCHILD domain ranged from 31 for the Positioning, transferring and mobility domain to 70 for the Comfort and emotions domain, with higher scores reflecting better health or easier care. The mean of the observed CHU9D utilities was 0.49. The distribution of CHU9D utilities was normal based on Shapiro–Wilk test (*p*-value 0.52). Supplement 1 shows histograms depicting the observed CHU9D utilities, CPCHILD total scores, and CPCHILD domain scores.

**Table 1:** Characteristics of participants

	Observations (*N* = 108)	Missing (*n*)
Age of the child (years)		1 (0.93%)
Mean (SD)	11.98 (4.20)	
Range	5-18	
Male children	66 (61.11%)	1 (0.93%)
Gross Motor Function Classification System (GMFCS) level		1 (0.93%)
Level IV	40 (37.04%)	
Level V	52 (48.15%)	
Not familiar with this classification or not sure	15 (13.89%)	
Highest education of the child has completed		1 (0.93%)
Kindergarten	11 (10.28%)	
1st to 6th grade	42 (39.26%)	
7th to 12th grade	31 (28.97%)	
Ungraded	23 (21.50%)	
Age of parents/caregivers (years)		7 (6.48%)
Mean (SD)	44.39 (9.28)	
Range	20-73	
Female parents/caregivers	100 (92.59%)	2 (1.85%)
Relationship with the child		5 (4.63%)
Biological parent	99 (91.67%)	
Foster parent	1 (0.93%)	
Guardian	2 (1.85%)	
Other, e.g. maternal grandmother	1 (0.93%)	
Work status		
Not working due to my child’s health	40 (37.04%)	
Not working for other reasons	8 (7.41%)	
Looking for work	2 (1.85%)	
Working full or part time	48 (44.44%)	
Full-time homemaker	12 (11.11%)	
Providing care 7 days a week	91 (84.26%)	5 (4.63%)
Highest education of the parents/caregivers		5 (4.63%)
Some high school or less	16 (14.81%)	
High school or diploma	14 (12.96%)	
Vocational school or some college	12 (11.11%)	
College or university degree	32 (29.36%)	
Professional or graduate degree	29 (26.85%)	
Perceived socioeconomic status		1 (0.93%)
Not at all well off	4 (3.70%)	
Not particularly well off	30 (27.78%)	
Fairly well off	58 (53.70%)	
Rather well off	12 (11.11%)	
Very well off	3 (2.78%)	
Observed CHU9D utilities		
Mean (SD)	0.485 (0.231)	
Range	0.001-0.989	
Observed CPCHILD total scores		
Mean (SD)	45.377 (13.498)	
Range	11.892-78.249	
Observed CPCHLD domain scores, mean (SD)		
Personal care/activities for daily living	32.246 (12.504)	
Positioning, transferring and mobility	30.912 (12.855)	
Comfort and emotions	70.121 (23.143)	
Communication and social interaction	40.366 (22.260)	
Health services utilisation and overall health	60.802 (22.152)	
Overall quality of life	55.000 (25.376)	

Table 2 presents the correlation coefficients of the CPCHILD domains and CHU9D utilities. The CPCHILD total score, CPCHILD Comfort and emotion, and CPCHILD overall quality of life score had moderate and significant correlations with the CHU9D utilities, with correlation coefficients of 0.56, 0.53, 0.50, respectively. In contrast, the Personal care/activities of daily living and the Positioning, transferring and mobility domains showed weak correlations with CHU9D utilities, with correlation coefficients below 0.35. These two domain scores were excluded from the predictor set 2 based on their weak correlations.

**Table 2:** Correlation coefficients between CHU9D utilities, CPCHILD total score, and CPCHILD domain scores

	Correlation coefficients	*p*-value
CPCHILD total score	0.5634	< 0.0001
CPCHILD personal care and activities of daily living domain score	0.3094	0.0011
CPCHILD positioning, transferring and mobility domain score	0.3494	0.0002
CPCHILD comfort and emotion domain score	0.5342	< 0.0001
CPCHILD communication and social interaction domain score	0.4058	< 0.0001
CPCHILD health service utilisation and overall health domain score	0.4289	< 0.0001
CPCHILD overall quality of life domain score	0.5026	< 0.0001

### Final model predictors

Age, gender, and GMFCS levels were not significant predictors and did not improve the predictive performance of the model. Therefore, these variables were excluded from all predictor sets. The remaining predictor(s) for *Predictor set 1* was the CPCHILD total score, *Predictor set 2* were the standardised domain scores of CPCHILD comfort and emotional, communication and social interaction, health, and quality of life that were statistically significant.

### Mapping regressions

Table 3 presents all measures of model performance using the full sample. Means of predicted CHU9D utilities were close to the observed utilities across all models. However, all regressions overestimated the lowest boundary and underestimated the highest boundary of the CHU9D utilities, resulting in the narrow range of predicted CHU9D than the observed values. The closest to the observed minimum utilities was the MM-estimate using Predictor set 2 (0.001 vs 0.058, Column 6). The highest predicted CHU9D utility was estimated from the GLM estimate with Gamma family and log link function using Predictor set 1 (0.983 vs 0.989, Column 7). Absolute differences between the predicted and observed CHU9D utilities were generally small across all regressions, except for the MM-estimator of the Predictor set 2.

**Table 3:** Mapping model performance using full samples

*N* = 108	Predictors	Differences between predicted and observed mean CHU9D utilities	Mean of predicted CHU9D utilities	Minimum predicted CHU9D utilities	Maximum predicted CHU9D utilities	Range of predicted CHU9D utilities	MAE	CCC	Recommended mapping model
Observed CHU9D utilities			0.485	0.001	0.989	0.988			
Predictor set 1: CPCHILD total score									
OLS		0.000	0.485	0.162	0.802	0.640	0.157^2^	0.482	
MM-estimator		-0.005	0.480	0.155	0.799	0.644	0.157^2^	0.483^c^	2nd choice
GLM Gaussian family log link		0.002	0.487	0.260	0.850	0.590	0.161^3^	0.442	
GLM Gaussian family logit link		0.002	0.487	0.190	0.787	0.596	0.157^2^	0.483^c^	
GLM Gamma family log link		0.003	0.488	0.217	0.983	0.766	0.162	0.492^b^	
GLM Gamma family logit link		0.005	0.490	0.165	0.818	0.653	0.156^1^	0.508^a^	1st choice
Predictor set 2: selected CPCHILD domain scores									
OLS	Comfort and emotion and Quality of life domain scores	0.000	0.485	0.108	0.706	0.598	0.155^3^	0.507	
MM-estimator	Comfort and emotion and Quality of life domain scores	-0.011	0.474	0.058	0.728	0.670	0.152^1^	0.531^c^	
GLM Gaussian family log link	Comfort and emotion and Quality of life domain scores	0.000	0.485	0.188	0.750	0.561	0.157	0.505	
GLM Gaussian family logit link	Comfort and emotion, Health and Quality of life domain scores	0.000	0.485	0.143	0.712	0.569	0.155^3^	0.518	
GLM Gamma family log link	Comfort and emotion, Health and Quality of life domain scores	0.003	0.488	0.153	0.905	0.752	0.153^2^	0.533^b^	2nd choice
GLM Gamma family logit link	Comfort and emotion, Health and Quality of life domain scores	0.008	0.493	0.101	0.790	0.689	0.152^1^	0.552^a^	1st choice

^1,2,3^Smallest, second, and third smallest MAE in Predictor set 1 and 2

^a,b,c^Largest, second, and third largest CCC in Predictor set 1 and 2

For the Predictor set 1, the GLM estimate with gamma family and logit link function outperformed other models based on its lowest MAE and highest CCC in both the full sample and cross-validation (Supplement 2) analyses. The second-best performing model in both the full sample and cross-validations was the MM-estimate. It had the second lowest MAE and a wider predictive range compared to the GLM estimate with Gaussian family and logit link (Table 3, Supplement 2).

For the Predictor set 2, the GLM estimate with gamma family and logit link function using scores from the CPCHILD comfort and emotion, health, and quality of life domains scores performed better than the other regression models. The second algorithm of choice was the GLM gamma family and log link using scores from comfort and emotion, health, and quality of life. Supplement 3 presents scatter plots that showcase the relationship between the observed and predicted CHU9D utilities of all mapping regressions.

Table 4 shows detailed regression results using the full sample. Algorithms of the most preferable mapping functions for each predictor set were presented as follows:

$$Predictor\,Set\,1\quad {\text{predicted}}\,{\text{CHU}}9{\text{D}}\,{\text{utilities}}\,\, = \,\,{\text{ln}}\frac{1}{{1 - \left( { - \,2.1839 + \left( {0.0471 \times {\text{CPCHILD}}_{{\text{total score}}} } \right)} \right)}}$$

$$Predictor\,set\,2\quad {\text{predicted}}\,{\text{CHU}}9{\text{D}}\,{\text{utility}}\, = \,\ln \left( {\frac{1}{{1 - \left( { - \,2.2743 + \left( {0.0112 \times {\text{CPCHILD}}_{{{\text{CE}}}} } \right) + \left( {0.0120 \times {\text{CPCHILD}}_{{\text{H}}} } \right) + \left( {0.0130 \times {\text{CPCHILD}}_{{{\text{QoL}}}} } \right)} \right)}}} \right),$$

where CPCHILD_CE_ is the CPCHILD Comfort and emotion standardised domain score, CPCHILD_H_ is the CPCHILD Health standardised domain score, and CPCHILD_QoL_ is the CPCHILD Quality of life standardised domain score.

**Table 4:** Regression outputs

	OLS	MM	GLM Gaussian family log link	GLM Gaussian family logit link	GLM Gamma family log link	GLM Gamma family logit link
Model 1: CPCHILD total score						
CPCHILD total score	0.0096*** (0.001)	0.0097*** (0.001)	0.0178*** (0.003)	0.0415*** (0.007)	0.02275** (0.003)	0.0471*** (0.007)
Constant	0.0472 (0.056)	0.0393 (0.059)	− 1.5588*** (0.142)	− 1.9406*** (0.316)	− 1.7965*** (0.159)	− 2.1839*** (0.274)
Model 2: selected CPCHILD domain scores						
CPCHILD comfort and emotions domain score	0.0036*** (0.001)	0.0036*** (0.001)	0.0108*** (0.003)	0.0173*** (0.005)	0.0078*** (0.002)	0.0112** (0.004)
CPCHILD quality of life domain score	0.0026** (0.001)	0.0034** (0.001)	0.0039* (0.002)	0.0110** (0.004)	0.0059* (0.002)	0.0130* (0.005)
CPCHILD health domain score					0.0050* (0.002)	0.0120** (0.004)
Constant	0.0851 (0.059)	0.0355 (0.052)	− 1.7380*** (0.19)	− 1.8988*** (0.327)	− 1.9387*** (0.162)	− 2.2743*** (0.256)

*t* statistics in parentheses

*p < 0.05, **p < 0.01, ***p < 0.001

## Discussion

Mapping algorithms play a crucial role in estimating health-related quality of life when MAUI is not administered. In the case of non-ambulatory children with CP, deriving CHU9D utilities from the CPCHILD is a valuable method for estimating the QALYs gain or loss resulting from interventions. Findings of this study can be used by researchers and clinicians who are interested in assessing utility values, providing valuable insights into healthcare decision-making and resource allocation in the context of cost-utility analysis.

The development of mapping algorithms adhered to the ISPOR mapping guidelines [11]. These guidelines provide a framework for the systematic development and validation of mapping algorithms. Moreover, this mapping study was reported in accordance with the MAP recommendations [12] to ensure transparency and consistency in reporting mapping process.

CPCHILD specifically focuses on children with greater physical impairments. It measures the health-related quality of life associated with functional limitations and well-being of these children from the perspective of their parents/caregivers. While some questions within the CPCHILD assess the ease of caregiving, the level of caregiving required is intrinsically linked to a child degree of impairment, which in turn has a direct influence on their well-being. This connection is further highlighted by a recent meta-analysis demonstrating a strong positive correlation between physical ability and quality of life in young individuals with CP [33]. This emphasises the importance of considering the caregiving aspect when evaluating quality of life of this population.

The relationship between physical impairment and quality of life is also evident in this study. Our study population reported substantially low quality of life. In particular, the average CHU9D utilities were almost half that of general young Australians (0.49 vs 0.78) [34]. The physical and/or psychological health of the parents/caregivers may affect the caregiving. However, this study did not investigate these impacts.

The selection of model predictors was based on careful consideration. The decision to exclude the CPCHILD Personal care/activities of daily living and Positioning, transferring and mobility domains was made due to their weak correlations. However, the CHU9D and CPCHILD total scores exhibited a moderate correlation. Moderate correlations were also observed between the CHU9D and CPCHILD Comfort and emotion and Quality of life domains (*ρ* 0.53 and 0.50, respectively). Correlation of CPQoL and CHU9D in the previous mapping study was 0.46 [10]. The moderate correlations are commonly observed in the mapping literature, as supported by previous studies [10, 26, 31].

All models overestimated the minimum value of CHU9D utilities and underestimated the maximum values, making the range of predicted CHU9D utilities narrower than the observed values. The narrow ranges of predicted values may raise concerns about the applicability of these mapping algorithms. However, this issue is not unique to this study, but has been evident in other mapping studies [10, 35, 36]. While Sharma et al. [35] attributed the narrow predictive range to the non-normal distribution of CHU9D utilities, the CHU9D utilities of our study are normally distributed. The relatively high MAEs (around 0.15 on the 1 utility scale) further support the presence of this issue. The sample size of this study is relatively small compared to other mapping studies which may have impact the model estimations. It is also possible that a small number of non-ambulatory participants with extreme utility values (approximately 10% of participants) influence the narrow ranges and high MAEs, but removing these participants did not resolve the issue (Supplement 4). To gain better understanding of the predicted range, a larger sample size with wider spread of utility scores would be beneficial.

Model performance indicators of our best performing models were not as good as the previously published mapping study that estimates CHU9D utilities from CPQoL [10]. The published mapping using selected CPQoL Child domain scores and GLM with Gaussian family logit link had MAE = 0.062 and CCC = 0.745 [10], whereas our best performing regression using selected CPCHILD domain scores and GLM with Gamma family logit link had MAE = 0.152 and CCC = 0.552. The high MAEs and CCCs observed in this study are difficult to explain, given that the statistical mapping methodologies employed were similar in both studies. The sample size in this current study was twice that of the previous one. The different goodness-of-fit findings between the two mapping studies could have resulted from differences in population characteristics, particularly as the population in this study had more profound impairment. Differences in classification systems (CPQoL vs CPCHILD) could also be a factor. Alternatively, these results may have stemmed from the reliance of proxy-reported information. The use of proxy-report could potentially introduce bias, as it may not fully capture the child’s subjective experiences and preferences, leading to discrepancies in the estimated utilities [37]. However, the previous mapping study [10] also utilised proxy-reported information. In CP research, the proxy-reported approach remains invaluable and is often the only feasible method. This study did not assess the bias of proxy-reporting on both measures, nor did it evaluate whether parents/caregivers adopted different perspectives while completing the questionnaires. Therefore, the extent to which proxy-reporting could lead to biased estimates remains unknown.

Despite facing challenges in participant recruitment during the Covid-19 lockdowns, this study was able to recruit over 100 participants, which is a significant achievement considering the pragmatic difficulties in recruiting large numbers of this specific population. While the sample size is relatively small compared to other mapping studies, it is larger than the mapping study of the CPQoL onto the CHU9D. In addition, previous CP trials have experienced low participant recruitment [10, 38]. To further validate and confirm the predictive ability of the suggested models, future studies with larger sample sizes are recommended.

## Conclusion

Twelve mapping models were developed to determine CHU9D utilities from either the CPCHILD total score or selected domain scores. Among these models, the regression model based on selected domain scores was recommended due to its superior performance indicators and predicted range, compared to the model based on CPCHILD total scores. The choice of model is contingent upon data availability. The use of alternative mapping algorithms should not lead to drastically different findings in economic evaluation, as their predictability is comparable. Future studies that employ our suggested models should investigate the impacts of utilising different algorithms. It should also be highlighted that mapping is considered as a second-best solution when direct MAUI data collection is not feasible.

To enhance the robustness of mapping CHU9D utilities from CPCHILD, future mapping studies should include a larger sample size, ensuring representation across the entire quality of life scale including those at the extreme ends of the scale, and incorporate an external dataset for validation.

## Supplementary Information

Below is the link to the electronic supplementary material.

Supplementary file1 (DOCX 68 KB)

Supplementary file2 (DOCX 22 KB)

Supplementary file3 (DOCX 242 KB)

Supplementary file4 (DOCX 15 KB)

## Data Availability

Collected surveys are not able to be shared.

## References

[1] Pharmaceutical Benefits Advisory Committee. (2016). Guidelines for preparing a submission to the Pharmaceutical Benefits Advisory Committee (version 5.0). https://pbac.pbs.gov.au/content/information/files/pbac-guidelines-version-5.pdf

[2] National Institute for Health and Clinical Excellence. (2013). Guide to the methods of technology appraisal 2013. https://www.nice.org.uk/process/pmg9/resources/guide-to-the-methods-of-technology-appraisal-2013-pdf-2007975843781 27905712

[3] Rosenbaum, P., Paneth, N., Leviton, A., Goldstein, M., Bax, M., Damiano, D., Dan, B., & Jacobsson, B. (2007). A report: The definition and classification of cerebral palsy April 2006. *Developmental Medicine & Child Neurology Supplement,**109*, 8–14. 17370477

[4] Tonmukayakul, U., Shih, S. T. F., Bourke-Taylor, H., Imms, C., Reddihough, D., Cox, L., & Carter, R. (2018). Systematic review of the economic impact of cerebral palsy. *Research in Developmental Disabilities,**80*, 93–101. 29981952 10.1016/j.ridd.2018.06.012

[5] Bahrampour, M., Downes, M., Scuffham, P. A., & Byrnes, J. (2021). Comparing multi-attribute utility instruments: CP-6D, a cerebral palsy specific instrument, vs AQoL-4D. *Expert Review of Pharmacoeconomics & Outcomes Research,**22*(2), 217–224. 33779449 10.1080/14737167.2021.1909477

[6] Mpundu-Kaambwa, C., Chen, G., Huynh, E., Russo, R., & Ratcliffe, J. (2018). A review of preference-based measures for the assessment of quality of life in children and adolescents with cerebral palsy. *Quality of Life Research,**27*(7), 1781–1799. 29569017 10.1007/s11136-018-1837-0

[7] Hu, J., Zhu, L., Han, B., Liu, Y., Xing, H., Kang, Q., & Jin, C. (2022). Utility estimations of different health states of patients with type I, II, and III spinal muscular atrophy in China: A mixed approach study with patient and proxy-reported data. *Frontiers in Public Health*. 10.3389/fpubh.2022.1054931 36605247 10.3389/fpubh.2022.1054931 PMC9809905

[8] Devlin, N., Pan, T., Kreimeier, S., Verstraete, J., Stolk, E., Rand, K., & Herdman, M. (2022). Valuing EQ-5D-Y: The current state of play. *Health and Quality of Life Outcomes,**20*(1), 105. 35794607 10.1186/s12955-022-01998-8 PMC9260978

[9] Ryan, J. M., McKay, E., Anokye, N., Noorkoiv, M., Theis, N., & Lavelle, G. (2020). Comparison of the CHU-9D and the EQ-5D-Y instruments in children and young people with cerebral palsy: A cross-sectional study. *BMJ Open,**10*(9), e037089. 32912983 10.1136/bmjopen-2020-037089 PMC7485239

[10] Tonmukayakul, U., Imms, C., Mihalopoulos, C., Reddihough, D., Carter, R., Mulhern, B., & Chen, G. (2020). Health-related quality of life and upper-limb impairment in children with cerebral palsy: Developing a mapping algorithm. *Developmental Medicine & Child Neurology,**62*(7), 854–860. 32064606 10.1111/dmcn.14488

[11] Wailoo, A. J., Hernandez-Alava, M., Manca, A., Mejia, A., Ray, J., Crawford, B., Botteman, M., & Busschbach, J. (2017). Mapping to estimate health-state utility from non-preference-based outcome measures: An ISPOR good practices for outcomes research task force report. *Value in Health,**20*(1), 18–27. 28212961 10.1016/j.jval.2016.11.006

[12] Petrou, S., Rivero-Arias, O., Dakin, H., Longworth, L., Oppe, M., Froud, R., & Gray, A. (2015). The MAPS reporting statement for studies mapping onto generic preference-based outcome measures: Explanation and elaboration. *PharmacoEconomics,**33*(10), 993–1011. 26232200 10.1007/s40273-015-0312-9

[13] Mortimer, D., & Segal, L. (2008). Comparing the incomparable? A systematic review of competing techniques for converting descriptive measures of health status into QALY-weights. *Medical Decision Making,**28*(1), 66–89. 18263562 10.1177/0272989X07309642

[14] Imms, C., Reilly, S., Carlin, J., & Dodd, K. (2008). Diversity of participation in children with cerebral palsy. *Developmental Medicine & Child Neurology,**50*(5), 363–369. 18355337 10.1111/j.1469-8749.2008.02051.x

[15] Harris, P. A., Taylor, R., Minor, B. L., Elliott, V., Fernandez, M., O’Neal, L., McLeod, L., Delacqua, G., Delacqua, F., Kirby, J., & Duda, S. N. (2019). The REDCap consortium: Building an international community of software platform partners. *Journal of Biomedical Informatics,**95*, 103208. 31078660 10.1016/j.jbi.2019.103208 PMC7254481

[16] Narayanan, U. G., Fehlings, D., Weir, S., Knights, S., Kiran, S., & Campbell, K. (2006). Initial development and validation of the Caregiver Priorities and Child Health Index of Life with Disabilities (CPCHILD). *Developmental Medicine & Child Neurology,**48*(10), 804–812. 16978459 10.1111/j.1469-8749.2006.tb01227.x

[17] Ramstad, K., Jahnsen, R., & Terjesen, T. (2016). Severe hip displacement reduces health-related quality of life in children with cerebral palsy. *Acta Orthopaedica,**88*(2), 205–10. 27892753 10.1080/17453674.2016.1262685 PMC5385117

[18] Zalmstra, T. A. L., Elema, A., Huizing, K., Reinders-Messelink, H. A., Putten, A., & vander. (2019). Longitudinal validation of the caregiver priorities and Child Health Index of Life with Disabilities in a Dutch Sample of Nonambulatory Children with Severe Disabilities. *Child Care Health and Development.,**45*, 409–416. 30870582 10.1111/cch.12663

[19] Narayanan, U. G., Weir, S., & Fehlings, D. (2007). The CPCHILD© Manual & Interpretation Guide.

[20] Stevens, K. (2012). Valuation of the Child Health Utility 9D Index. *PharmacoEconomics,**30*(8), 729. 22788262 10.2165/11599120-000000000-00000

[21] Xiong, X., Li, H., Herd, D., Borland, M. L., Davidson, A., Hearps, S., Lee, K. J., Dalziel, S. R., Dalziel, K., Cheek, J. A., & Babl, F. E. (2023). Cost-effectiveness of prednisolone to treat bell palsy in children. *Neurology*. 10.1212/WNL.0000000000207284 37072220 10.1212/WNL.0000000000207284 PMC10264054

[22] Stevens, K. (2011). Assessing the performance of a new generic measure of health-related quality of life for children and refining it for use in health state valuation. *Applied Health Econnomics and Health Policy,**9*(3), 157–169. 10.2165/11587350-000000000-00000 21506622

[23] Ratcliffe, J., Flynn, T., Terlich, F., Stevens, K., Brazier, J., & Sawyer, M. (2012). Developing adolescent specific health state values for economic evaluation: An application of profile case best worst scaling to the Child Health Utility-9D. *PharmacoEconomics,**30*(8), 713–727. 22788261 10.2165/11597900-000000000-00000

[24] Ratcliffe, J., Huynh, E., Chen, G., Stevens, K., Swait, J., Brazier, J., Sawyer, M., Roberts, R., & Flynn, T. (2016). Valuing the Child Health Utility 9D: Using profile case best worst scaling methods to develop a new adolescent specific scoring algorithm. *Social Science & Medicine,**157*, 48–59. 27060541 10.1016/j.socscimed.2016.03.042

[25] Hinkle, D. E., Wieersma, W., & Jurs, S. G. (2003). *Applied statistics for the behavioural sciences*. Houghton Mifflin.

[26] Mpundu-Kaambwa, C., Chen, G., Russo, R., Stevens, K., Petersen, K. D., & Ratcliffe, J. (2017). Mapping CHU9D Utility Scores from the PedsQLTM 4.0 SF-15. *PharmacoEconomics,**35*(4), 453–467. 27928758 10.1007/s40273-016-0476-y

[27] Mukuria, C., Rowen, D., Harnan, S., Rawdin, A., Wong, R., Ara, R., & Brazier, J. (2019). An updated systematic review of studies mapping (or cross-walking) measures of health-related Quality of Life to generic preference-based measures to generate utility values. *Applied Health Economics and Health Policy,**17*(3), 295–313. 30945127 10.1007/s40258-019-00467-6

[28] Rand, R. W. (2012). *Introduction to robust estimation and hypothesis testing* (3rd ed.). Academic Press.

[29] Sun, Q., & Zhang, F. (2021). Current status of research on the mapping function of health utility values in the Asia Pacific region: A systematic review. *Value in Health Regional Issues,**24*, 224–239. 33894684 10.1016/j.vhri.2020.12.008

[30] Barnhart, H. X., Haber, M., & Song, J. (2002). Overall concordance correlation coefficient for evaluating agreement among multiple observers. *Biometrics,**58*(4), 1020–1027. 12495158 10.1111/j.0006-341X.2002.01020.x

[31] Chen, G., Stevens, K., Rowen, D., & Ratcliffe, J. (2014). From KIDSCREEN-10 to CHU9D: Creating a unique mapping algorithm for application in economic evaluation. *Health and Quality of Life Outcomes,**12*(1), 134. 25169558 10.1186/s12955-014-0134-z PMC4243726

[32] StataCorp. (2019). *Stata statistical software: Release 16*. StataCorp LLC.

[33] Makris, T., Dorstyn, D., & Crettenden, A. (2021). Quality of life in children and adolescents with cerebral palsy: A systematic review with meta-analysis. *Disability and Rehabilitation,**43*(3), 299–308. 31180733 10.1080/09638288.2019.1623852

[34] Le, L. K., Richards-Jones, S., Chatterton, M. L., Engel, L., Lawrence, D., Stevenson, C., Pepin, G., Ratcliffe, J., Sawyer, M., & Mihalopoulos, C. (2021). Australian adolescent population norms for the Child Health Utility Index 9D-results from the young minds matter survey. *Quality of Life Research,**30*(10), 2895–2906. 33999321 10.1007/s11136-021-02864-8 PMC8126511

[35] Sharma, R., Gu, Y., Sinha, K., Aghdaee, M., & Parkinson, B. (2019). Mapping the Strengths and Difficulties Questionnaire onto the Child Health Utility 9D in a large study of children. *Quality of Life Research,**28*(9), 2429–2441. 31154585 10.1007/s11136-019-02220-x

[36] Furber, G., Segal, L., Leach, M., & Cocks, J. (2014). Mapping scores from the Strengths and Difficulties Questionnaire (SDQ) to preference-based utility values. *Quality of Life Research,**23*(2), 403–411. 23943259 10.1007/s11136-013-0494-6

[37] Wallander, J. L., & Koot, H. M. (2016). Quality of life in children: A critical examination of concepts, approaches, issues, and future directions. *Clinical Psychology Review,**45*, 131–43. 26911191 10.1016/j.cpr.2015.11.007

[38] Imms, C., Wallen, M., Elliott, C., Hoare, B., Greaves, S., Randall, M., & Orsini, F. (2022). Implications of providing wrist-hand orthoses for children with cerebral palsy: Evidence from a randomised controlled trial. *Disability Rehabilitation,**45*, 1–11. 35649128 10.1080/09638288.2022.2079734

