# Correction: Development of algorithms for estimating the Child Health Utility 9D from Caregiver Priorities and Child Health Index of Life with Disabilities

**DOI:** 10.1007/s11136-024-03771-4

**Published:** 2024-09-12

**Authors:** Utsana Tonmukayakul, Kate Willoughby, Cathrine Mihalopoulos, Dinah Reddihough, Brendan Mulhern, Rob Carter, Suzanne Robinson, Gang Chen

**Affiliations:** 1 Deakin Health Economics, Institute for Health Transformation, Faculty of Health, Deakin University, Geelong, VIC Australia; 2 Orthopaedic Department, The Royal Children’s Hospital, Parkville, Melbourne, VIC Australia; 3 Murdoch Children’s Research Institute, Parkville, Melbourne, VIC Australia; 4 Division of Health Economics, Public Health and Preventive Medicine, Monash University, Melbourne, VIC Australia; 5 Centre of Health Economics Research and Evaluation, University of Technology Sydney, Haymarket, Sydney, NSW Australia; 6 Centre for Health Economics, Monash Business School, Monash University, Melbourne, VIC Australia

**Correction to: Quality of Life Research (2024) 33:1881–1891**

10.1007/s11136-024-03661-9

In the original published article, the title was incorrectly given as “Development of algorithms for estimating the Child Health Utility 9D from Caregiver Priorities and Child Health Index of Life with Disability” but should have been “Development of algorithms for estimating the Child Health Utility 9D from Caregiver Priorities and Child Health Index of Life with Disabilities”.

The original article has been corrected.

