# Supplementary material for: Development of algorithms for estimating the Child Health Utility 9D from Caregiver Priorities and Child Health Index of Life with Disabilities
Source: Qual Life Res. 2024 May 3;33(7):1881–91. doi: 10.1007/s11136-024-03661-9 (PMC11176203; doi:10.1007/s11136-024-03661-9)
Supplement: Supplementary file 1 — Supplementary file1 (DOCX 68 KB) [file 11136_2024_3661_MOESM1_ESM.docx]

**Supplement 1 Histograms of CHU9D utilities, CPCHILD total scores and CPCHILD standardised domain scores**

**Figure 3a CHU9D utilities**

**Figure 3b CPCHILD total score**

**Figure 3c CPCHILD Personal care/activities of daily living domain score**

**Figure 3d CPCHILD positioning, transferring and mobility domain score**

**Figure 3e CPCHILD Comfort and emotion domain score**

**Figure 3f CPCHILD Communication and social interaction domain score**

**Figure 3g CPCHILD Health domain score**

**Figure 3h CPCHILD Quality of life domain score**
