# Supplementary material for: Development of algorithms for estimating the Child Health Utility 9D from Caregiver Priorities and Child Health Index of Life with Disabilities
Source: Qual Life Res. 2024 May 3;33(7):1881–91. doi: 10.1007/s11136-024-03661-9 (PMC11176203; doi:10.1007/s11136-024-03661-9)
Supplement: Supplementary file 2 — Supplementary file2 (DOCX 22 KB) [file 11136_2024_3661_MOESM2_ESM.docx]

**Supplement 2 Mapping model performance results from cross-validation techniques**

|  | **Predicted variables** | **Differences between predicted and observed mean CHU9D utilities** | **Mean of predicted CHU9D utilities** | **Minimum predicted CHU9D utilities** | **Maximum predicted CHU9D utilities** | **Range of predicted CHU9D utilities** | **MAE** | **CCC** | **Recommended mapping model** |
| --- | --- | --- | --- | --- | --- | --- | --- | --- | --- |
| Observed CHU9D utilities |  |  | 0.485 | 0.001 | 0.989 | 0.988 |  |  |  |
| **5-FOLD CROSS VALIDATION** | | | | | | | | | |
| ***Predictor set 1: CPCHILD total score*** | | | | | | | | | |
| OLS |  | 0.001 | 0.486 | 0.147 | 0.834 | 0.687 | 0.162^3^ | 0.464^b^ |  |
| MM-estimator |  | -0.004 | 0.481 | 0.142 | 0.832 | 0.690 | 0.159^2^ | 0.461 |  |
| GLM Gaussian family log link |  | 0.003 | 0.488 | 0.248 | 0.908 | 0.660 | 0.165 | 0.422 |  |
| GLM Gaussian family logit link |  | 0.003 | 0.488 | 0.172 | 0.817 | 0.645 | 0.158^1^ | 0.464^b^ | 2^nd^ choice |
| GLM Gamma family log link |  | 0.005 | 0.490 | 0.218 | 1.000 | 0.782 | 0.171 | 0.477^b^ |  |
| GLM Gamma family logit link |  | 0.007 | 0.492 | 0.169 | 0.829 | 0.660 | 0.159^2^ | 0.499^a^ | 1^st^ choice |
| ***Predictor set 2: selected CPCHILD domain scores*** | | | | | | | | | |
| OLS | Comfort and emotion &  Quality of life domain scores | 0.001 | 0.486 | 0.147 | 0.834 | 0.687 | 0.155^1^ | 0.479 |  |
| MM-estimator | Comfort and emotion &  Quality of life domain scores | -0.011 | 0.474 | 0.025 | 0.745 | 0.721 | 0.163 | 0.505^b^ |  |
| GLM Gaussian family log link | Comfort and emotion &  Quality of life domain scores | 0.001 | 0.486 | 0.163 | 0.805 | 0.642 | 0.162 | 0.476 |  |
| GLM Gaussian family logit link | Comfort and emotion, Health &  Quality of life domain scores | 0.000 | 0.485 | 0.110 | 0.743 | 0.633 | 0.164 | 0.492 |  |
| GLM Gamma family log link | Comfort and emotion, Health &  Quality of life domain scores | 0.003 | 0.488 | 0.141 | 1.00 | 0.859 | 0.160^3^ | 0.495^c^ | 2^nd^ choice |
| GLM Gamma family logit link | Comfort and emotion, Health &  Quality of life domain scores | 0.008 | 0.493 | 0.090 | 0.862 | 0.771 | 0.157^2^ | 0.522^a^ | 1^st^ choice |
| **80 RANDOM SAMPLES** | | | | | | | | | |
| ***Predictor set 1: CPCHILD total score*** | | | | | | | | | |
| OLS |  | -0.005 | 0.480 | 0.164 | 0.770 | 0.606 | 0.164^2^ | 0.448 |  |
| MM-estimator |  | -0.008 | 0.477 | 0.153 | 0.773 | 0.620 | 0.163^1^ | 0.453^c^ | 2^nd^ choice |
| GLM Gaussian family log link |  | -0.003 | 0.482 | 0.253 | 0.821 | 0.568 | 0.168 | 0.412 |  |
| GLM Gaussian family logit link |  | -0.003 | 0.482 | 0.194 | 0.759 | 0.565 | 0.164^2^ | 0.445 |  |
| GLM Gamma family log link |  | -0.002 | 0.483 | 0.212 | 0.940 | 0.729 | 0.167^3^ | 0.460 ^b^ |  |
| GLM Gamma family logit link |  | 0.000 | 0.485 | 0.167 | 0.792 | 0.625 | 0.164^2^ | 0.472^a^ | 1^st^ choice |
| ***Predictor set 2: selected CPCHILD domain scores*** | | | | | | | | | |
| OLS | Comfort and emotion &  Quality of life domain scores | -0.005 | 0.480 | 0.113 | 0.712 | 0.598 | 0.156^3^ | 0.509 |  |
| MM-estimator | Comfort and emotion &  Quality of life domain scores | -0.015 | 0.470 | 0.053 | 0.748 | 0.695 | 0.153^1^ | 0.540^b^ |  |
| GLM Gaussian family log link | Comfort and emotion &  Quality of life domain scores | -0.004 | 0.481 | 0.193 | 0.754 | 0.562 | 0.161 | 0.494 |  |
| GLM Gaussian family logit link | Comfort and emotion, Health &  Quality of life domain scores | -0.004 | 0.481 | 0.142 | 0.720 | 0.578 | 0.157 | 0.522^c^ |  |
| GLM Gamma family log link | Comfort and emotion, Health &  Quality of life domain scores | -0.001 | 0.484 | 0.155 | 0.923 | 0.769 | 0.156^3^ | 0.522^c^ | 2^nd^ choice |
| GLM Gamma family logit link | Comfort and emotion, Health &  Quality of life domain scores | 0.003 | 0.488 | 0.108 | 0.790 | 0.682 | 0.155^2^ | 0.542^a^ | 1^st^ choice |
| **50 RANDOM SAMPLES** | | | | | | | | | |
| ***Predictor set 1: CPCHILD total score*** | | | | | | | | | |
| OLS |  | -0.034 | 0.451 | 0.110 | 0.763 | 0.653 | 0.147^2^ | 0.558 |  |
| MM-estimator |  | -0.032 | 0.453 | 0.106 | 0.771 | 0.664 | 0.147^2^ | 0.562^c^ | 2^nd^ choice |
| GLM Gaussian family log link |  | -0.032 | 0.453 | 0.218 | 0.814 | 0.596 | 0.151 | 0.529 |  |
| GLM Gaussian family logit link |  | -0.033 | 0.452 | 0.156 | 0.759 | 0.603 | 0.148^3^ | 0.557 |  |
| GLM Gamma family log link |  | -0.031 | 0.454 | 0.185 | 0.915 | 0.729 | 0.153 | 0.570^b^ |  |
| GLM Gamma family logit link |  | -0.030 | 0.455 | 0.142 | 0.780 | 0.638 | 0.146^1^ | 0.572^a^ | 1^st^ choice |
| ***Predictor set 2: selected CPCHILD domain scores*** | | | | | | | | | |
| OLS | Comfort and emotion &  Quality of life domain scores | -0.034 | 0.451 | 0.064 | 0.686 | 0.621 | 0.154^3^ | 0.541 |  |
| MM-estimator | Comfort and emotion &  Quality of life domain -scores | -0.045 | 0.440 | 0.030 | 0.689 | 0.660 | 0.153^2^ | 0.554^c^ |  |
| GLM Gaussian family log link | Comfort and emotion &  Quality of life domain scores | -0.033 | 0.452 | 0.160 | 0.731 | 0.571 | 0.155 | 0.531 |  |
| GLM Gaussian family logit link | Comfort and emotion, Health &  Quality of life domain scores | -0.033 | 0.455 | 0.125 | 0.687 | 0.562 | 0.155 | 0.536 |  |
| GLM Gamma family log link | Comfort and emotion, Health &  Quality of life domain scores | -0.029 | 0.456 | 0.154 | 0.825 | 0.671 | 0.151^1^ | 0.571^a^ | 2^nd^ choice |
| GLM Gamma family logit link | Comfort and emotion, Health &  Quality of life domain scores | -0.018 | 0.467 | 0.098 | 0.772 | 0.674 | 0.151^1^ | 0.565^b^ | 1^st^ choice |

Note: ^1,2,3^ indicates the smallest, second, and third smallest MAE in Predictor set 1 and 2.

^a,b,c^ indicates the largest, second, and third largest CCC in Predictor set 1 and 2.
