# Supplementary material for: Development of algorithms for estimating the Child Health Utility 9D from Caregiver Priorities and Child Health Index of Life with Disabilities
Source: Qual Life Res. 2024 May 3;33(7):1881–91. doi: 10.1007/s11136-024-03661-9 (PMC11176203; doi:10.1007/s11136-024-03661-9)
Supplement: Supplementary file 3 — Supplementary file3 (DOCX 242 KB) [file 11136_2024_3661_MOESM3_ESM.docx]

**Supplement Scatter plots of observed and predicted CHU9D utilities**

Figure 1: Observed and predicted CHU9D utilities OLS using CPCHILD total score as a predictor

Figure 2: Observed and predicted CHU9D utilities MM-estimator using CPCHILD total score as a predictor

Figure 3: Observed and predicted CHU9D utilities GLM Gaussian family log link using CPCHILD total score as a predictor

Figure 4: Observed and predicted CHU9D utilities GLM Gaussian family logit link using CPCHILD total score as a predictor

Figure 5: Observed and predicted CHU9D utilities GLM Gamma family log link using CPCHILD total score as a predictor

Figure 6: Observed and predicted CHU9D utilities from GLM Gamma family logit link using CPCHILD total score as a predictor

Figure 7: Observed and predicted CHU9D utilities from OLS using CPCHILD comfort & emotion and quality of life domain scores as predictors

Figure 8: Observed and predicted CHU9D utilities from MM-estimator using CPCHILD comfort & emotion and quality of life domain scores as predictors

Figure 9: Observed and predicted CHU9D utilities from GLM Gaussian family using CPCHILD comfort & emotion and quality of life domain scores as predictors

Figure 10: Observed and predicted CHU9D utilities from GLM Gaussian family using CPCHILD comfort & emotion and quality of life domain scores as predictors

Figure 11: Observed and predicted CHU9D utilities from GLM Gamma family log link using CPCHILD comfort & emotion, health, and quality of life domain scores as predictors

Figure 12: Observed and predicted CHU9D utilities from GLM Gamma family logit link using CPCHILD comfort & emotion, health, and quality of life domain scores as predictors
