# Supplementary material for: Development of algorithms for estimating the Child Health Utility 9D from Caregiver Priorities and Child Health Index of Life with Disabilities
Source: Qual Life Res. 2024 May 3;33(7):1881–91. doi: 10.1007/s11136-024-03661-9 (PMC11176203; doi:10.1007/s11136-024-03661-9)
Supplement: Supplementary file 4 — Supplementary file4 (DOCX 15 KB) [file 11136_2024_3661_MOESM4_ESM.docx]

**Supplement 4 Mapping model performance using samples with CHU9D utilities between 0.1 and 0.9**

| **N = 99** | **Predictor** | **Mean of predicted CHU9D utilities** | **Minimum predicted CHU9D utilities** | **Maximum predicted CHU9D utilities** | **Range of predicted CHU9D utilities** | **Difference from observed mean** |
| --- | --- | --- | --- | --- | --- | --- |
| Observed CHU9D utilities |  | 0.488 | 0.113 | 0.888 | 0.775 |  |
| **Predictor set 1: CPCHILD total score** | | | | | | |
| OLS |  | 0.484 | 0.237 | 0.727 | 0.490 | 0.286 |
| MM-estimator |  | 0.480 | 0.222 | 0.733 | 0.510 | 0.281 |
| GLM Gaussian family log link |  | 0.486 | 0.299 | 0.757 | 0.459 | 0.288 |
| GLM Gaussian family logit link |  | 0.485 | 0.252 | 0.720 | 0.469 | 0.287 |
| GLM Gamma family log link |  | 0.486 | 0.279 | 0.803 | 0.524 | 0.288 |
| GLM Gamma family logit link |  | 0.485 | 0.242 | 0.732 | 0.490 | 0.287 |
| **Predictor set 2: selected CPCHILD domain scores** | | | | | | |
| OLS | Comfort and emotion &  Quality of life domain scores | 0.481 | 0.195 | 0.648 | 0.452 | 0.283 |
| MM-estimator | Comfort and emotion &  Quality of life domain scores | 0.471 | 0.135 | 0.676 | 0.540 | 0.272 |
| GLM Gaussian family log link | Comfort and emotion &  Quality of life domain scores | 0.483 | 0.245 | 0.672 | 0.428 | 0.284 |
| GLM Gaussian family logit link | Comfort and emotion, Health &  Quality of life domain scores | 0.481 | 0.210 | 0.653 | 0.443 | 0.283 |
| GLM Gamma family log link | Comfort and emotion, Health &  Quality of life domain scores | 0.485 | 0.214 | 0.755 | 0.541 | 0.286 |
| GLM Gamma family logit link | Comfort and emotion, Health &  Quality of life domain scores | 0.486 | 0.167 | 0.706 | 0.539 | 0.287 |
